# A Protocol for Preconceptional Screening of Consanguineous Couples Using Whole Exome Sequencing

**DOI:** 10.3389/fgene.2021.685123

**Published:** 2021-10-25

**Authors:** Carolina Maria de Araújo dos Santos, Ana Helena Heller, Heloisa Barbosa Pena, Sérgio Danilo Junho Pena

**Affiliations:** ^1^GENE – Núcleo de Genética Médica, Belo Horizonte, Brazil; ^2^Laboratório de Genômica Clínica, Faculdade de Medicina da Universidade Federal de Minas Gerais, Belo Horizonte, Brazil; ^3^Departamento de Bioquímica e Imunologia, Universidade Federal de Minas Gerais, Belo Horizonte, Brazil

**Keywords:** consanguinity, preconceptional genetic diagnosis, carrier screening, whole exome sequencing, genetic counseling

## Abstract

Genetic studies performed in consanguineous couples suggest that the reproductive risk that distinguish them from other couples in the general population is related to autosomal recessive (AR) diseases. This risk is scattered among the thousands of known and potential AR diseases. Thus, for effective preconceptional screening of consanguineous couples it is necessary a test that encompasses the largest number of genes possible. For that reason, we decided to create a protocol based on whole exome sequencing (WES). We sequenced completely the exomes of 39 consanguineous couples at high coverage (∼100×). Applying bioinformatics filters, we could detect genetic variants that were simultaneously present in both members of the couple in all genes listed in the Clinical Genomics Database as causally related to AR diseases. Shared variants were then assessed for pathogenicity. For non-truncating variants (missense and in-frame indels) we considered as pathogenic or likely pathogenic only the variants included as such in the ClinVar database. Shared truncating variants (frameshift, non-sense, and canonical splice variants) were considered likely pathogenic when loss-of-function was a known mechanism of disease. The 39 consanguineous cases included two couples with a coefficient of genetic relationship (CGR) of 0.25, 26 couples with a CGR of 0.125, three couples with a CGR of 0.0625 and eight couples with a CGR of 0.03125. In 21 of the 39 couples (53.8%) we ascertained sharing of heterozygosity for at least one variant considered pathogenic or likely pathogenic for an AR disease. In eight couples we found sharing of heterozygosity for at least two pathogenic variants. Once the specific pathogenic variant was identified, it became possible for the couple to undergo prenatal diagnosis or, if desired, preimplantation genetic diagnosis (PGD) involving *in vitro* fertilization and embryo screening. In conclusion, our results demonstrate that preconceptional screening by WES is a useful new procedure that should be incorporated in the genetic counseling of all consanguineous couples.

## Introduction

Autosomal recessive (AR) inherited disorders can be a major cause of morbidity and mortality (Bundey and Alam, 1993). The occurrence of these disorders can significantly increase in the offspring of consanguineous couples. Approximately 1.1 billion people live currently in countries where consanguineous marriages are customary, and among them, one in every three marriages is between cousins (Hamamy et al., 2011). Consequently, in many locations, consanguineous couples and their offspring represent a significant proportion of any genetic counselor’s case load. The identification of the risks for these couples should enable them to consider reproductive choices to prevent the birth of affected children, including prenatal diagnosis and preimplantation genetic testing.

In theory, genetic counseling could offer an acceptable approach to reduce the burden of recessively inherited disease. However, genetic counseling alone can only present risk estimates and does not offer to the consanguineous couple the possibility of taking practical measures to avoid genetic disease in their future children, since knowledge of the exact gene and variant are necessary for that. Moreover, there seems to exist a lack of standardization of genetic services. A questionnaire sent to certified genetics counselors and medical geneticists in the United States has revealed a wide variation in the risk figures quoted to consanguineous couples about their risk of having offspring with birth defects and intellectual deficiency (Bennett et al., 1999).

Advances in screening couples for heterozygosity over the past decades offer now the possibility of testing populations for all known severe recessive genetic disorders (Antonarakis, 2019). However, to offer effective pre-conceptive screening for consanguineous couples it is not necessary to identify all the pathogenic variants for which each member of a couple is a carrier, being sufficient to identify only which pathogenic variants are shared by them.

Fridman et al. (2021) studied 6,447 exome sequences of healthy, genetically unrelated Europeans from Holland and Estonia and calculated that almost all individuals (>85%) carry at least one pathogenic or likely pathogenic (PLP) variant, with an average of at least 1.3 PLPs for a severe AR disorder and 2.2 PLPs for any AR disorder. Their data did not allow estimation of the upper bound, but they felt it unlikely that the number of PLPs would exceed 8 per individual. If novel AR genes that have not yet been discovered contribute less PLPs than those that have been discovered recently, then the upper bound for the estimate would be more in the range of 4–5 PLPs per individual. They estimate first-cousin consanguineous couples to be at 16 times higher risk to conceive a child with an AR disorder compared to non-consanguineous couples. This translates to 3,400 newborns with a severe AR disorder per 100,000 births for first cousins (3.4%). These estimates are compatible with the calculation that couples who are first cousins show an extra 2–3% increased risk of having children with AR genetic problems (Hamamy et al., 2011). As expected, the risks gradually decreased for more distant relationships and the risk for third cousins was similar to that for non-consanguineous couples (Fridman et al., 2021).

It is important to keep in mind that this risk is diffuse, scattered among the thousands of potential AR diseases. Thus, to prospectively detect consanguineous couples at increased risk, it is necessary to carry out a genetic test with high coverage and high sensitivity, which specifically examines whether both members of the couple are simultaneously carriers of the same pathogenic or probably pathogenic variant, in heterozygosis. The ideal tests for this purpose appear to be whole genome sequencing (WGS) and whole exome sequencing (WES). Several articles have been written providing proof of concept for the use of WES in preconceptional screening for AR diseases (Makrythanasis et al., 2014; Teeuw et al., 2014; Sallevelt et al., 2017; Kirk et al., 2019; Monies et al., 2019).

Challenges remain regarding how to identify which shared genetic variants are capable of causing disease in homozygosity. It is important to avoid ambiguous results and incidental findings by keeping the analysis absolutely focused on the only element that distinguishes consanguineous couples from other couples: the increased risk of producing sons or daughters with an AR disease due to homozygosis. It is also essential to work only with diseases that have a known molecular basis, in order to be able to implement practical preventive measures.

We designed and wish to present a protocol for preconceptional screening of consanguineous couples using WES. According to this protocol, we first sequence the exome of the two members of the consanguineous couple, and then use bioinformatics to filter the genetic information to detect heterozygous genetic variants that are common to both. Our targets are the causative genes of all AR diseases obtained through a survey updated monthly in the Clinical Genomic Database of the National Human Genome Research Institute (2021). Presently, 2,846 genes related to AR diseases (including all AD-AR genes) are listed there.

## Materials and Methods

### Patients

We collected buccal swabs from 39 consanguineous couples who presented for genetic counseling at GENE – Núcleo de Genética Médica de Minas Gerais in Belo Horizonte, Brazil. DNA was extracted using a modified salting out procedure (Miller et al., 1988).

The Research Ethics Committee of the Hospital das Clínicas of the Universidade Federal de Minas Gerais approved the study protocol. Informed consent was obtained according to current ethical and legal guidelines. The study was conducted in accordance with the Declaration of Helsinki.

### Whole Exome Sequencing

After DNA extraction and pertinent technical procedures, exome enrichment by the Agilent SureSelect XT V6 was performed at Theragen Bio (Seongnam-si, Gyeonggi-do, Republic of Korea), with a capture greater than 50 Mb. The library was sequenced in an Illumina NovaSeq 6000 instrument following the manufacturer’s specifications and resulted in over 92 million readings, with more than 14 Gigabases of sequence data, allowing an average coverage greater than 100×. Signal processing and base identification (base calling) was performed with the FastQC software, followed by the alignment of the tested exome to the reference human genome (hg19) using the BWA software. Variants were determined by the GATK Unified Genotyper software following parameters specified by the Broad Institute, which developed the software.

### Bioinformatics Analysis

Sequenced genes were filtered for rare variants (allele frequency < 0.01) utilizing databases such as 1000 Genomes Phase 3, NHLBI Exome Sequencing Project (ESP6500), Single Nucleotide Polymorphism database (dbSNP141) and gnomAD database, using the Mendel, MD software developed in-house (Cardenas et al., 2017) and the ENLIS Genome Research software (Enlis Genomics, Berkeley, CA, United States). To analyze the impact of the candidate variants we used the software Alamut Visual version 2.11.0 (Interactive Biosoftware, Paris, France, which showed the alignment of orthologous genes, the gnomAD frequencies and the ClinVar classification. Variants were also analyzed with the Franklin software (The Genoox Platform, 2021). To define what constitutes a pathogenic variant, we used the criteria of the American College of Medical Genetics and Genomics (Richards et al., 2015). Thus, only missense and in-frame indel variants already classified as such in the ClinVar database (ClinVar, 2021) are considered pathogenic or probably pathogenic. Truncating variants that are not in the ClinVar database are considered probably pathogenic when loss-of-function is a known disease mechanism.

## Results and Discussion

The 39 consanguineous cases included two couples with a coefficient of genetic relationship (CGR) of 0.25, 26 couples with a CGR of 0.125, three couples with a CGR of 0.0625, and eight couples with a CGR of 0.03125 (Table 1). In 21 of these 39 couples (53.8%) we ascertained sharing of heterozygosis for at least one variant considered pathogenic for an AR disease (Table 1). In eight couples we found sharing of heterozygosity for at least two pathogenic variants.

**TABLE 1:** Results of the search for the same pathogenic and likely pathogenic variants in both members of 39 consanguineous couples.

Degree of consanguinity	CGR	PLP in common	Disease	OMIM	Gene	Variant
Uncle-niece	0.25	Yes	Pyruvate kinase deficiency in red blood cells	266200	*PKLR*	c.1456C > T p.(Arg486Trp)
Double first cousins	0.25	Yes	Breast and colorectal cancer, susceptibility to Intellectual deficiency, autosomal recessive 3 Deafness, autosomal recessive 1A	604373 608443 220290	*CHEK2* *CC2D1A* *GJB2*	c.975 + 1G > C c.1357-2A > C c.109G > A p.(Val37Ile)
First cousins	0.125	Yes	Nemaline myopathy 2, autosomal recessive asphyxiating thoracic dystrophy 3	256030 613091	*NEB* *DYNC2H1*	c.1258-2A > G c.6047A > G p.(Tyr2016Cys)
1First cousins	0.125	Yes	Dyskeratosis congenita, autosomal recessive 3	613988	*WRAP53*	c.1564dup p.(Ala522Glyfs*8)
First cousins	0.125	Yes	Glycogen storage disease Ib cranioectodermal dysplasia 1	232220 218330	*SLC37A4* *IFT122*	c.935_936del p.(Thr312Serfs*13) c.1301-1G > C
First cousins	0.125	Yes	Homocystinuria Adrenal hyperplasia, congenital	236200 201910	*CBS CYP21A2*	c.833T > C p.(Ile278Thr) c.955C > T p.(Gln319*)
First cousins	0.125	Yes	Mitochondrial complex V deficiency, nuclear type 2 citrullinemia	614052 215700	*TMEM70 ASS1*	c.317-2A > G c.323G > T p.(Arg108Leu)
First cousins	0.125	Yes	Stargardt disease 1	248200	*ABCA4*	c.2588G > C p.(Gly863Ala)
First cousins	0.125	Yes	Papillon-lefevre syndrome	245000	*CTSC*	c.194_197dup p.(Tyr67Profs*11)
First cousins	0.125	Yes	Galactosemia	230400	*GALT*	c.563A > G p.(Gln188Arg)
First cousins	0.125	Yes	Spondylocostal dysostosis 6	616566	*RIPPLY2*	c.238A > T p.(Arg80*)
First cousins	0.125	Yes	Thyroid dyshormonogenesis 6	607200	*DUOX2*	c.2428G > T p.(Glu810*)
First cousins	0.125	Yes	Sickle cell anemia hyperlipoproteinemia, type I	603903 238600	*HBB LPL*	c.20A > T p.(Glu7Val) c.953A > G p.(Asn318Ser)
First cousins	0.125	Yes	Microcephaly 1 deafness 1A	251200 220290	*MCPH1 GJB2*	c.2145G > A p.(Trp715*) c.35del p.(Gly12Valfs*2)
First cousins	0.125	Yes	Cystathionine beta-synthase deficiency familial adenomatous polyposis 2	236200 608456	*CBS MUTYH*	c.833T > C p.(Ile278Thr) c.721C > T p.(Arg241Trp)
First cousins	0.125	No				
First cousins	0.125	No				
First cousins	0.125	No				
First cousins	0.125	No				
First cousins	0.125	No				
First cousins	0.125	No				
First cousins	0.125	No				
Half uncle-niece	0.125	No				
First cousins	0.125	No				
First cousins	0.125	No				
First cousins	0.125	No				
First cousins	0.125	No				
First cousins	0.125	No				
First cousins once removed	0.0625	Yes	Acyl-Coa Dehydrogenase, very long-chain deficiency	201475	*ACADVL*	c.685C > T p.(Arg229*)
First cousins once removed	0.0625	Yes	Amelogenesis imperfecta, type IJ	617297	*ACPT*	c.945dup p.(Glu316*)
First cousins once removed	0.0625	No				
Second cousins	0.03125	Yes	Deafness, autosomal recessive 77	613079	*LOXHD1*	c.2047 + 1G > A
Second cousins	0.03125	Yes	Fetal akinesia deformation sequence 1	208150	*SCN4A*	c.1173del p.(Phe392Serfs*12)
Second cousins	0.03125	Yes	Joubert Syndrome, Type 13	614173	*TCTN1*	c.1775_1778del p.(Val592_593delins30)
Second cousins	0.03125	Yes	Inflammatory bowel disease 28, early onset	613148	*IL10RA*	c.349C > T p.(Arg117Cys)
Second cousins	0.03125	No				
Second cousins	0.03125	No				
Second cousins	0.03125	No				
Second cousins	0.03125	No				

Except for 25 first cousin couples, the number of the other types of relationship were small. This possibly can explain why the data do not show a decrease in the proportion of positive cases in first cousins once removed (CGR = 0.0625). We should mention that these coefficients of genetic relationships are based on family history and should be considered estimates. A major drawback of pedigree-based calculations is the absolute requirement for a correct pedigree structure, which in practice may be unreliable, or incomplete.

If we limit our analysis to the 25 first cousin couples, we can observe that 13 of them (52%) have one or more shared pathogenic or likely pathogenic (PLP) variant. Although 26 couples are not a large number, this percentage is more than double the rate of 20.9–24.9% at risk couples (ARC) estimated by Fridman et al. (2021) for first cousins, based on simulated consanguineous matings. We can derive mathematically the relationship of the rate of ARCs to the PLPs for a given CGR with the formula:


$$1-{\left(1-\mathrm{CGR}\right)}^{\text{PLP}}=\mathrm{ARC}$$

For first cousins (CGR = 0.125) and ARCs in the range of 20.9–24.9%, we can use the formula to calculate PLPs in the range of 1.75–2.14, which is compatible with the calculation in the European population of 2.2 PLPs for any AR disorder. However, for the ARC of 0.52 in our sample of first cousin couples we would obtain a larger value of 5.5 PLPs for any AR disease. However, one should not place emphasis on this discrepancy, since we cannot rule out that it was originated from random variation or from ascertainment biases.

If now we focus our attention on the nature of the PLP variants encountered in common in our first cousin group we observe that 11/20 (55%) are truncating loss-of-function (LoF) variants, which is compatible with the data of Fridman et al. (2021) who found that more than half of the PLPs (55.2 and 59.1% in the Dutch and Estonian cohorts) were truncating loss-of-function (LoF) rare variants. However, our data differs from theirs since 64% of our truncating loss-of-function (LoF) variants were described in Clinvar, while none in their study were. Since our numbers are small, this discrepancy could be aleatory.

Once the specific pathogenic variant was identified, it became possible for the couple to undergo prenatal diagnosis or, if desired, preimplantation genetic diagnosis (PGD) involving *in vitro* fertilization and embryo screening.

Our results demonstrate that preconceptional screening by WES is a useful new procedure that should be incorporated in the genetic counseling of all consanguineous couples. However, it is still not perfect for several reasons:

First, the number of AR diseases known is still relatively small. According to the calculations of Bamshad et al. (2019), if we assume that each candidate gene underlies a single Mendelian condition (MC), there are circa 1.5–3 times as many novel genes (4,450–10,467) for MCs yet to be discovered as there are genes (3,519) known already to underlie an MC. If we extrapolate that the same proportion of these genes underlie multiple MCs as is the case for known genes for MCs (i.e., 16% underlie two MCs, 4.7% underlie three, 1.8% underlie four, etc.), we can predict that a minimum of 6,100–14,400 MCs remain to be discovered. And these figures are still an underestimate of the number of unsolved MCs because the authors did not account for the fact that mutant phenotypes for over half (∼12,000) of all protein-coding mouse genes have yet to be assessed.

Second, our ClinVar criteria for identification of disease-causing non-truncating genetic variants are by necessity very conservative, because we cannot risk burdening consanguineous couples with false-positive variants. Even so, the possibility of false-positive finding exists because of the unpredictable contingency of incomplete penetrance (Cooper et al., 2013).

Third, it is important to remember that WES does not detect all genetic variants present in an individual. Among the situations in which WES may not be able to identify a pathogenic variant, we can highlight: (a) mutations by deletion or duplication of the entire gene or part of it; microsatellite expansion mutations (dynamic mutations); (c) diseases associated with mutations in two different genes (digenic inheritance); (d) mutations that are not located in an exon (for example, a pathogenic mutation in a promoter or intronic region), (e) mutations in genes whose exons are captured in low efficiency by the currently available exonic selection kits; (f) mutations in a gene that is very similar to other genes (paralogs) in the human genome; and (h) Mutations caused by the insertion of a transposing element.

Because of these caveats, a negative result in the search for common PLPs in consanguineous couples should not be interpreted as the absence of genetic risks. In fact, couples that do not initiate reproduction immediately are offered the possibility of annual revisions of the exome data, to permit incorporation of new knowledge in human genomics. Also, the finding of a disease-causing variant in a couple does not mean that it is the only genetic risk present. The observation that some consanguineous couples occasionally have more than one shared pathogenic gene means that even couples who have already had a child diagnosed with an AR disease should undergo pre-conceptional screening by WES because of the possibility of other shared pathogenic variants.

## Conclusion

Although there is considerable room for progress and inevitable intrinsic uncertainty (Wray and Loo, 2015), our results demonstrate that preconceptional screening by WES is already a viable and useful new procedure that should be incorporated in the genetic counseling routine of all consanguineous couples.

This protocol can also be used to assist family planning for couples who belong to the same ethnic group (Ashkenazi Jews, Sephardic Jews, Mediterraneans, Sub-Saharan Africans, members of endogamous communities, etc.), whose increased risk for group-specific AR diseases is well known.

## Data Availability Statement

The original contributions presented in the study are included in the article/supplementary material, further inquiries can be directed to the corresponding author.

## Ethics Statement

The studies involving human participants were reviewed and approved by Research Ethics Committee of the Hospital das Clínicas of the Universidade Federal de Minas Gerais. The patients/participants provided their written informed consent to participate in this study.

## Author Contributions

CS and AH were responsible for the general conduction of the study, contact with the consanguineous couples, preparing reports, and participation in writing the manuscript. HP was responsible for technical procedures of extraction of DNA, quantitative measurements of purity, and conduction of the study. SP conceived the protocol, analyzed the exome results, and participated in writing the manuscript. All authors contributed to the article and approved the submitted version.

## Conflict of Interest

The authors declare that the research was conducted in the absence of any commercial or financial relationships that could be construed as a potential conflict of interest.

## Publisher’s Note

All claims expressed in this article are solely those of the authors and do not necessarily represent those of their affiliated organizations, or those of the publisher, the editors and the reviewers. Any product that may be evaluated in this article, or claim that may be made by its manufacturer, is not guaranteed or endorsed by the publisher.
